# Mood Predicts Response to Placebo CPAP

**DOI:** 10.1155/2012/404196

**Published:** 2012-09-03

**Authors:** Carl J. Stepnowsky, Wei-Chung Mao, Wayne A. Bardwell, José S. Loredo, Joel E. Dimsdale

**Affiliations:** ^1^Department of Medicine, University of California, San Diego, La Jolla, CA 92093, USA; ^2^Health Services Research & Development Service, Veterans Affairs San Diego Healthcare System, San Diego, CA 92161, USA; ^3^Department of Psychiatry, Tri-Service General Hospital, Taipei, Taiwan; ^4^Department of Psychiatry, University of California, San Diego, La Jolla, CA 92093, USA

## Abstract

*Study Objectives*. Continuous positive airway pressure (CPAP) therapy is efficacious for treating obstructive sleep apnea (OSA), but recent studies with placebo CPAP (CPAP administered at subtherapeutic pressure) have revealed nonspecific (or placebo) responses to CPAP treatment. This study examined baseline psychological factors associated with beneficial effects from placebo CPAP treatment. *Participants*. Twenty-five participants were studied with polysomnography at baseline and after treatment with placebo CPAP. *Design*. Participants were randomized to either CPAP treatment or placebo CPAP. Baseline mood was assessed with the Profile of Mood States (POMS). Total mood disturbance (POMS-Total) was obtained by summing the six POMS subscale scores, with Vigor weighted negatively. The dependent variable was changed in apnea-hypopnea index (ΔAHI), calculated by subtracting pre- from post-CPAP AHI. Negative values implied improvement. Hierarchical regression analysis was performed, with pre-CPAP AHI added as a covariate to control for baseline OSA severity. *Results*. Baseline emotional distress predicted the drop in AHI in response to placebo CPAP. Highly distressed patients showed greater placebo response, with a 34% drop (i.e., improvement) in AHI. *Conclusion*. These findings underscore the importance of placebo-controlled studies of CPAP treatment. Whereas such trials are routinely included in drug trials, this paper argues for their importance even in mechanical-oriented sleep interventions.

## 1. Introduction

Placebo responses remain one of the great mysteries of medicine. While a clinical benefit of treatment (any treatment) is always a “plus” for the patient, such placebo responses can complicate clinical trials. This paper examines some placebo response characteristics in patients being treated with continuous positive airway pressure (CPAP) for obstructive sleep apnea (OSA). 

OSA is a potentially devastating illness that involves sleep fragmentation and associated hypoxia, affecting up to 9% of middle-aged adults and higher percentages in the elderly [1–3]. The pathological consequences of upper airway obstruction during sleep may lead to cardiovascular complications, marked psychological distress, and impairment in daytime performance and cognitive functioning [4–9]. The most commonly used treatment is nasal CPAP [10, 11].

CPAP has been efficacious in improving many outcome measurements, especially in patients with severe OSA [12–15]. However, some researchers have reported inconsistencies in improvement, for example, a nonspecific (or placebo) effect of CPAP in mild OSA patients [16, 17] or disparate findings between subjective (e.g., self-report questionnaires) and objective measurements (e.g., neuropsychological assessment and ambulatory blood pressure monitoring) in response to CPAP [18–21]. The mechanical rationale for CPAP (i.e., that air pressure will keep the airway open) is so powerful that placebo studies of CPAP are still fairly unusual. Some studies have employed oral placebos [18], but a small number of research groups have been applying CPAP administered at subtherapeutic pressures [14, 16, 20, 22] in the belief that the apparatus at the bedside would provide a “more powerful” placebo. 

Previous studies from our research group have examined this placebo effect of CPAP treatment. Loredo et al. reported that CPAP and placebo CPAP had comparable effects on sleep quality (as assessed by sleep architecture, sleep efficiency, total sleep time, and time awake after sleep onset), but placebo CPAP had little effect on AHI [22]. Bardwell et al. found no significant difference after treatment in neuropsychological assessment between placebo and CPAP groups [23]. Yu et al. concluded that the effect of CPAP treatment on mood symptoms in apneic patients could be a placebo effect [24]. All of these studies found obvious treatment effects in the CPAP groups in a variety of objective or subjective domains. However, the statistical significance of many of these effects declined or disappeared completely after comparison with placebo CPAP groups. Such findings document the importance of including a placebo-controlled group in CPAP treatment studies. 

Given these findings, we wondered what factors distinguished between placebo “responders” and “nonresponders.” Such information may help guide clinical treatment options or the design of future clinical research. We hypothesized that emotional distress level predicts placebo response to CPAP treatment.

## 2. Materials and Methods

### 2.1. Participants and Procedures

Participants with a history suggestive of sleep apnea were recruited by advertising and word of mouth. None of the participants had ever experienced or seen a CPAP apparatus prior to this study. To qualify, they had to be 100% to 200% of ideal body weight as determined by Metropolitan Life Insurance tables [25]. Although OSA is more common among the obese, participants >200% of ideal body weight were excluded because of the possibility of confounding by other conditions associated with morbid obesity. Participants were also excluded if they had a history of other major medical or psychiatric problems or hypertension greater than 180/110 mm Hg. Those with hypertension were slowly withdrawn from their medications at least two weeks before participation in order to eliminate possible drug effects on sleep. All participants gave written consent for the study, which was approved by the Institutional Review Board at the University of California, San Diego.

All participants were studied with polysomnography (PSG). PSG included central and occipital electroencephalogram (EEG), submental electromyogram (EMG), nasal/oral airflow using a thermistor, thoracic and abdominal excursions with respitrace respiratory inductive plethysmography, and bilateral tibialis anterior EMG. Oxygen saturation levels were monitored using a pulse oximeter (Biox 3740; Ohmeda: Louisville, CO) and were analyzed using computer software (Profox; Escondido, CA).

Sleep recordings were scored according to the criteria of Rechtshaffen and Kales [26]. Apneas were defined as decrements in airflow >90% from baseline for a period >10 seconds. Hypopneas were defined as decrements in airflow >50% but <90% from baseline for a period >10 seconds. Potential participants who showed predominant central apneas (>50% of total apneas) were excluded from this study. The number of apneas and hypopneas per hour was calculated to obtain the apnea-hypopnea index (RDI). 

Participants with an AHI >10 were classified as having OSA and were then randomized to receive CPAP or placebo CPAP. Both the patients and researchers were blinded to the treatment modes. To examine characteristics of individuals whose AHI dropped on placebo, we combined data from 2 trials that used common procedures. Both studies assigned patients to receive double-blind either true CPAP or placebo CPAP. Study 1 examined the effects of one week of CPAP at pressure <2 cm H_2_O; Study 2 examined the effects of 2 weeks of CPAP at pressure <1 cm H_2_O. 

In patients randomized to active treatment, conventional manual overnight CPAP titration during PSG was employed to provide optimal effective CPAP pressure to minimize OSA [22–24]. These active treatment patients are not the focus of this paper; instead, the paper examines the AHI response of patients randomized to placebo treatment. 

The placebo CPAP group underwent a mock-CPAP titration night similar in all aspects to the CPAP treatment group except that CPAP pressure at the nose was maintained at less than 2 cm of H_2_O in Study 1 and less then 1 cm of H_2_O in Study 2 (these levels are well below those required to eliminate nocturnal respiratory events). These participants used a modified CPAP machine at the bedside in conjunction with a specially designed CPAP mask containing multiple one-quarter inch drill holes to insure adequate gas exchange and patient comfort. This placebo-CPAP system is a modification of that described by Farre and colleagues [27]. Participants were instructed to use the nasal CPAP unit (DeVilbiss Horizon; Somerset, PA) at home during sleep. 

**Figure 1:**
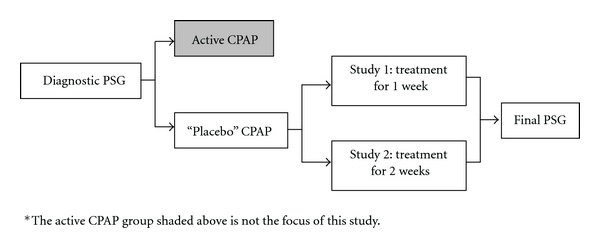
Diagram of study design*.

Each CPAP unit had a hidden compliance clock that measured the amount of time that the unit was powered “on.” Compliance was measured on each of the protocol nights and was defined as the average number of hours the machine was powered on per night over the course of the protocol. It was presumed that whenever the unit was on, it was being used. The participants returned for another night of PSG after seven days of placebo CPAP use at home for Study 1 and after 14 days for Study 2. Compliance was averaged over the treatment interval. Twenty-five participants received placebo CPAP treatment in these two studies (16 from Study 1 and 9 from Study 2). This study design is summarized schematically in Figure 1.

Mood of participants was assessed with the Profile of Mood States (POMS) [28] at baseline (prior to randomization) and after the participants returned for PSG followup (one week in Study 1 and two weeks in Study 2). The POMS is a well-established factor-analytically derived measure of psychological distress for which high levels of reliability and validity have been documented. The POMS consists of 65 adjectives that are rated on a 0 to 4 scale. Higher scores indicate greater levels of distress. Data from this instrument can be consolidated into 6 dimensions of mood: tension-anxiety, depression-dejection, fatigue-inertia, confusion-bewilderment, vigor-activity, and anger-hostility. The POMS has been used in a variety of chronically ill and normal populations [29, 30], including OSA patients [31, 32]. Because of the small sample size, we reduced the number of independent variables by using “POMS-Total” to represent the total mood distress. This was obtained by summing the six POMS subscale scores, with Vigor weighted negatively.

### 2.2. Statistical Methods

The dependent variable was the change in AHI (ΔAHI), calculated by subtracting the pre-CPAP AHI from the post-CPAP AHI. Negative values implied improvement in the severity of sleep apnea. A *t*-test was done to compare the difference between Study 1 and Study 2 in Baseline POMS-Total and ΔAHI. Groups representing high and low emotional distress were created by median split on the Baseline POMS-Total score. A *t*-test was used to compare the compliance with placebo CPAP and the change in AHI in the two distress groups. Because the severity of OSA may affect the treatment response, the pre-CPAP AHI was controlled as a covariate in the analyses.  

A hierarchical regression analysis procedure was used to predict ΔAHI. In step  1, a dummy variable was entered to identify which study the subjects participated in. In step  2, pre-CPAP AHI was added as a covariate to control for baseline OSA severity. Finally, in step  3, baseline POMS-Total was added to the model. Adjusted *R* ^2^ represents *R* ^2^ after accounting for sample size. All analyses were performed using SPSS 17.0 software.

## 3. Results

Participant characteristics are shown in Table 1. Three participants were women, representing the higher OSA prevalence in men. Participants were middle-aged (age range 33–60 years) and mildly obese. At baseline, participants were considered to be mildly to moderately emotionally distressed (POMS-Total = 37.7 ± 31.3). The level of distress showed improvement after placebo CPAP treatment, from 37.7 to 20.5 (*P* = 0.025).  Table 2 shows partial correlations between the six POMS subscales and ΔAHI while controlling for pre-CPAP AHI. All POMS subscales except Vigor were inversely correlated with ΔAHI, implying that greater distress at baseline was associated with greater improvement in AHI when treated with placebo CPAP.

Median splitting on the POMS baseline resulted in 12 high-distress participants (Baseline POMS-Total = 57.8 ± 25) and 13 low-distress participants (Baseline POMS-Total = 12.5 ± 13.4). There was no statistically or clinically significant difference in CPAP compliance between these two groups (5.64 versus 5.77 hrs, *P* = 0.77). The high-distress group showed significant improvement after placebo CPAP and dropped their AHI from 46.3 to 30.6 (*P* = 0.004), while the low-distress group did not change their AHI after placebo treatment (RDI from 44.1 to 46.5, *P* = 0.483).

**Figure 2:**
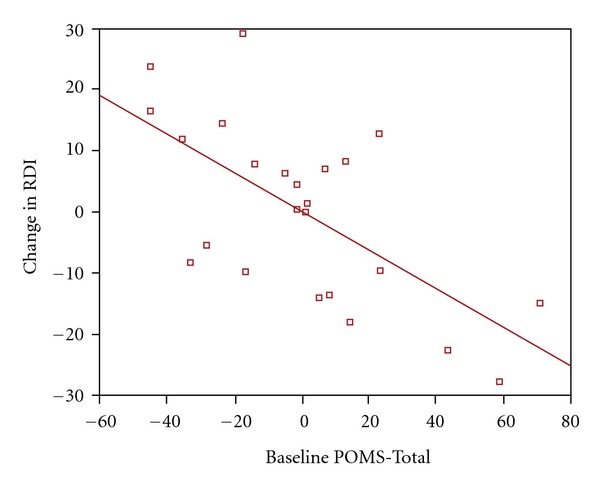
Partial regression plot of change in AHI versus baseline POMS-Total after controlling for baseline AHI.

We also examined the data using a hierarchical regression analysis (see Table 3). In step  1, no group differences (Study 1 versus Study 2) were found for ΔAHI (adjusted *R* ^2^ = 0.04, *F* = 0.853, *P* = 0.365). In step  2, study and pre-CPAP AHI did not predict ΔRDI in response to placebo CPAP (adjusted *R* ^2^ = 0.02, *F* = 1.198, *P* = 0.321). In step  3, the full model was highly significant (adjusted *R* ^2^ = 0.353, *F* = 5.361, *P* = 0.007). Baseline POMS-Total independently accounted for a significant amount of variance in ΔRDI beyond that accounted for by study and pre-CPAP AHI (adjusted Δ *R* ^2^ = 0.337, *F* = 8.344, *P* = 0.002). Baseline POMS-Total was negatively associated with ΔRDI (*P* = 0.001); (i.e., greater distress at baseline was associated with a greater decrease in AHI after treatment with placebo CPAP).  Figure 2 shows the partial regression plot between ΔRDI and Baseline POMS-Total after controlling for baseline AHI.

CPAP responders and nonresponders were identified via median splitting on AHI. *t*-tests were run on the sleep variables to investigate whether sleep variables differed between the groups. The groups differed on arousal index (25.2 ± 15.2 versus 12.6 ± 6.4; *P* = 0.005) and percentage of stage 1 (13.8 ± 8.2 versus 8.5 ± 2.6; *P* = 0.019) (nonresponder versus responder, resp.), such that nonresponders had a higher arousal index and more stage 1 sleep than responders. The groups did not differ on sleep efficiency, stage 2, deep sleep, REM sleep, or amount of awake after sleep onset. 

## 4. Discussion

The double-blind randomized controlled trial has been viewed as the strongest method of validating therapy [33]. Researchers have a number of options for their choice of control interventions, including placebo, usual care, or some form of active intervention [34]. An ideal placebo is an intervention that closely mimics the active intervention so that a comparison can be made [34]. Our placebo CPAP groups used what most would consider subtherapeutic CPAP pressure, with holes in the mask to further limit its effectiveness. This allowed duplication of the obvious aspects of the CPAP intervention yet ostensibly did away with the benefit that results from fully effective treatment. Such a study design allowed us to investigate the placebo effect. 

The overall placebo group, although still having substantial respiratory disturbance following treatment, showed reduction in AHI from 45.2 to 38.9 (ΔRDI ranged from −39.5 to 25). The high-distress group showed significant improvement after placebo CPAP treatment, dropping 33.9% in AHI (from 46.3 to 30.6, *P* = 0.004). 

The CPAP pressure used in our placebo may have had a partial therapeutic effect, which suggests a dose-response nature of CPAP treatment. However, this level of pressure is much lower than what would be expected to significantly reduce the AHI. Previous reports showed that a minimum CPAP pressure of 4 to 6 cm of H_2_O was needed to control AHI [35], and 4 cm H_2_O was required just to prevent snoring in nonapneics [36]. Although the possibility must be considered that CPAP at 1-2 cm of H_2_O pressure might not be an “absolutely inactive” placebo, previous reports do not support this interpretation of our results.

A central observation of this study was that emotional distress predicted the drop in AHI in response to placebo CPAP. People who experienced greater benefit from placebo CPAP were those who were more distressed prior to treatment. In fact, the most obvious improvement in AHI occurred in the high emotional distress group; AHI levels were lowered by 34%. A comparison of POMS-Total before and after placebo-CPAP treatment suggests that such placebo effects also resulted in reduced emotional distress. These findings suggest a strong psychological impact of CPAP treatment.

By investigating the placebo effects in this model, we might better understand the specific components of the total treatment effect. This could help future CPAP studies to control more of the confounders, thus allowing better assessment of the specific benefits of treatment. Not including a placebo-control group or ignoring the strong psychological impact from CPAP treatment itself may help to explain some of the inconsistencies in the literature regarding the benefits of CPAP.

Possible explanations for the placebo effects we observed include the obvious expectations of benefit from the CPAP treatment. Wearing sophisticated CPAP equipment could have comforted the patients, particularly those patients who were more distressed prior to treatment. Nonspecific beneficial effects are commonly seen in the initial period after introduction of a therapy; whether such benefits persist over time remains to be seen.

This study reminds us that the placebo effect can be very powerful, at least in the short term. Little is known about the characteristics of placebo responders. In our sample of sleep apneics, we found that the placebo effect may be influenced by emotional distress. These observations suggest the importance of placebo-controlled studies of CPAP treatment. If these observations generalize to other reactions to placebo treatment, it would suggest that the strongest placebo responders are those who enter clinical studies with high levels of emotional distress.

The limitations of this study include the following: (1) its small sample size and short period of treatment and (2) the variables for placebo effect (POMS and AHI). It is not clear whether such findings would generalize to other outcome measurements. Nevertheless, typical CPAP intervention studies in OSA patients are usually small, with treatment periods varying from as few as two days to several months [37–39]. In addition, we combined participants from two studies, having slightly different treatment protocols, but study protocol was not a significant variable in the analyses. While distressed patients may be very appreciative of attention, it is still hard to understand the mechanism linking high distress to a strong beneficial placebo response. This was an unanticipated finding. However, given that it was revealed in two separate study samples, the observation needs to be considered. It is certainly not surprising that people who were more distressed/depressed improved their morale more than those who were less distressed. This is, after all, the essence of regression to the mean. Might there, however, be some underexplored links between depressed mood and respiratory disturbance itself? There is a long history of investigating the effects of antidepressants of varying classes as treatments for sleep apnea. The observation linking distress and AHI improvement with placebo CPAP appears robust, observed across two separate samples, but the mechanism remains to be determined. 

Medicine is increasingly aware of the power of the placebo. This study examined characteristics of placebo responders to a highly specific intervention-continuous positive airways pressure treatment for obstructive sleep apnea. As newer treatments for OSA become available, consideration of the design issues that we raise may help guide future studies.

## Figures and Tables

**Table 1:** Participant characteristics (Mean ± SD).

*N*	25
Gender (M/F)	22/3
Age	48.8 ± 8.4
BMI	29.7 ± 5.4
Hypertension (no/yes)	19/6
Pre-CPAP AHI (range)	45.2 ± 28.3 (10.9–109.2)
Post-CPAP AHI (range)	38.9 ± 31.6 (5.8–118.4)
Baseline POMS-Total	37.7 ± 31.3
Follow-up POMS-Total	20.5 ± 26.4

BMI: body mass index (kg/m^2^), CPAP: continuous positive airway pressure, RDI: Apnea-hypopnea index, POMS: profile of mood states.

**Table 2:** Partial correlation coefficients (*r*) (after controlling pre-CPAP AHI).

	ΔRDI	Tension	Depression	Fatigue	Confusion	Vigor
Tension	−.57**					
Depression	−.67**	.76**				
Fatigue	−.43*	.62**	.47*			
Confusion	−.52*	.60**	.69**	.49*		
Vigor	.19	−.21	−.14	−.45*	−.09	
Anger	−.50*	.75**	.72**	.57**	.63**	−.01

*P < 0.05, **P < 0.01.

**Table 3:** Prediction of change in AHI (hierarchical regression analysis).

Variable	*B*	SE *B *	*β*	*R* ^2^	Adjusted *R* ^2^	*F*	*P*
Step 1				.036	−.006	.853	.365
Study*	6.233	6.7	.189				.365
Step 2				.098	.016	1.198	.321
Study	13.102	8.691	.397				.146
Pre-CPAP AHI	−.185	.150	−.325				.230
Step 3				.434	.353	5.361	.007
Study	2.270	7.689	.069				.771
Pre-CPAP AHI	−.012	.131	−.021				.929
Baseline POMS-Total	−.342	.097	−.639				.002

*“Study” is a dummy variable indicating from which protocol the subject was drawn.

## References

[1] Ancoli-Israel S, Kripke DF, Klauber MR, Mason WJ, Fell R, Kaplan O (1991). Sleep-disordered breathing in community-dwelling elderly. *Sleep*.

[2] Young T, Palta M, Dempsey J, Skatrud J, Weber S, Badr S (1993). The occurrence of sleep-disordered breathing among middle-aged adults. *New England Journal of Medicine*.

[3] Orr WC (1986). Sleep apnea, hypoxemia, and cardiac arrhythmias. *Chest*.

[4] Lanfranchi P, Somers VK (2001). Obstructive sleep apnea and vascular disease. *Respiratory Research*.

[5] Kraiczi H, Peker Y, Caidahl K, Samuelsson A, Hedner J (2001). Blood pressure, cardiac structure and severity of obstructive sleep apnea in a sleep clinic population. *Journal of Hypertension*.

[6] Engleman HM, Kingshott RN, Martin SE, Douglas NJ (2000). Cognitive function in the sleep apnea/hypopnea syndrome (SAHS). *Sleep*.

[7] Day R, Gerhardstein R, Lumley A, Roth T, Rosenthal L (1999). The behavioral morbidity of obstructive sleep apnea. *Progress in Cardiovascular Diseases*.

[8] Akashiba T, Kawahara S, Kosaka N (2002). Determinants of chronic hypercapnia in Japanese men with obstructive sleep apnea syndrome. *Chest*.

[9] Green DE, Schulman DA (2010). Obstructive sleep apnea and cardiovascular disease. *Current Treatment Options in Cardiovascular Medicine*.

[10] Sullivan CE, Issa FG, Berthon-Jones M, Eves L (1981). Reversal of obstructive sleep apnoea by continuous positive airway pressure applied through the nares. *The Lancet*.

[11] Epstein LJ, Kristo D, Strollo PJ (2009). Clinical guideline for the evaluation, management and long-term care of obstructive sleep apnea in adults. *Journal of Clinical Sleep Medicine*.

[12] McArdle N, Douglas NJ (2001). Effect of continuous positive airway pressure on sleep architecture in the sleep apnea-hypopnea syndrome: a randomized controlled trial. *American Journal of Respiratory and Critical Care Medicine*.

[13] Barbé F, Mayoralas LR, Duran J (2001). Treatment with continuous positive airway pressure is not effective in patients with sleep apnea but no daytime sleepiness: a randomized, controlled trial. *Annals of Internal Medicine*.

[14] Jenkinson C, Davies RJO, Mullins R, Stradling JR (1999). Comparison of therapeutic and subtherapeutic nasal continuous positive airway pressure for obstructive sleep apnoea: a randomised prospective parallel trial. *The Lancet*.

[15] McFadyen TA, Espie CA, McArdle N, Douglas NJ, Engleman HM (2001). Controlled, prospective trial of psychosocial function before and after continuous positive airway pressure therapy. *European Respiratory Journal*.

[16] Barnes M, Houston D, Worsnop CJ (2002). A randomized controlled trial of continuous positive airway pressure in mild obstructive sleep apnea. *American Journal of Respiratory and Critical Care Medicine*.

[17] Engleman HM, Martin SE, Deary IJ, Douglas NJ (1997). Effect of CPAP therapy on daytime function in patients with mild sleep apnoea/hypopnoea syndrome. *Thorax*.

[18] Engleman HM, Kingshott RN, Wraith PK, Mackay TW, Deary IJ, Douglas NJ (1999). Randomized placebo-controlled crossover trial of continuous positive airway pressure for mild sleep apnea/hypopnea syndrome. *American Journal of Respiratory and Critical Care Medicine*.

[19] Dimsdale JE, Loredo JS, Profant J (2000). Effect of continuous positive airway pressure on blood pressure: a placebo trial. *Hypertension*.

[20] Henke KG, Grady JJ, Kuna ST (2001). Effect of nasal continuous positive airway pressure on neuropsychological function in sleep apnea-hypopnea syndrome: a randomized, placebo-controlled trial. *American Journal of Respiratory and Critical Care Medicine*.

[21] Redline S, Adams N, Strauss ME, Roebuck T, Winters M, Rosenberg C (1998). Improvement of mild sleep-disordered breathing with CPAP compared with conservative therapy. *American Journal of Respiratory and Critical Care Medicine*.

[22] Loredo JS, Ancoli-Israel S, Dimsdale JE (1999). Effect of continuous positive airway pressure vs placebo continuous positive airway pressure on sleep quality in obstructive sleep apnea. *Chest*.

[23] Bardwell WA, Ancoli-Israel S, Berry CC, Dimsdale JE (2001). Neuropsychological effects of one-week continuous positive airway pressure treatment in patients with obstructive sleep apnea: a placebo-controlled study. *Psychosomatic Medicine*.

[24] Yu BH, Ancoli-Israel S, Dimsdale JE (1999). Effect of CPAP treatment on mood states in patients with sleep apnea. *Journal of Psychiatric Research*.

[25] (1983). 1983 metropolitan height and weight tables. *Stat Bull Metrop Life Found*.

[26] Rechstschaffen A, Kales A (1968). *Manual of Standardized Terminology, Techniques and Scoring Systems for Sleep Stages of Human Subjects*.

[27] Farré R, Hernández L, Montserrat JM, Rotger M, Ballester E, Navajas D (1999). Sham continuous positive airway pressure for placebo-controlled studies in sleep apnoea. *The Lancet*.

[28] McNair DM, Lorr M, Droppleman LF (1992). *POMS Manual: Profile of Mood States*.

[29] Spiegel D, Bloom JR, Yalom I (1981). Group support for patients with metastatic cancer. A randomized prospective outcome study. *Archives of General Psychiatry*.

[30] Taylor SE, Lichtman RR, Wood JV (1984). Attributions, beliefs about control, and adjustment to breast cancer. *Journal of Personality and Social Psychology*.

[31] Dickel MJ, Mosko SS (1990). Morbidity cut-offs for sleep apnea and periodic leg movements in predicting subjective complaints in seniors. *Sleep*.

[32] Mosko S, Zetin M, Glen S (1989). Self-reported depressive symptomatology, mood ratings, and treatment outcome in sleep disorders patients. *Journal of Clinical Psychology*.

[33] Kaptchuk TJ (1998). Powerful placebo: the dark side of the randomised controlled trial. *The Lancet*.

[34] Vickers AJ, de Craen AJM (2000). Why use placebos in clinical trials? A narrative review of the methodological literature. *Journal of Clinical Epidemiology*.

[35] Berry RB, Block AJ (1984). Positive nasal airway pressure eliminates snoring as well as obstructive sleep apnea. *Chest*.

[36] Issa FG, Sullivan CE (1984). Upper airway closing pressures in snorers. *Journal of Applied Physiology Respiratory Environmental and Exercise Physiology*.

[37] Valencia-Flores M, Bliwise DL, Guilleminault C, Cilveti R, Clerk A (1996). Cognitive function in patients with sleep apnea after acute nocturnal nasal continuous positive airway pressure (CPAP) treatment: sleepiness and hypoxemia effects. *Journal of Clinical and Experimental Neuropsychology*.

[38] Scheltens P, Visscher F, van Keimpema ARJ, Lindeboom J, Taphoorn MJB, Wolters EC (1991). Sleep apnea syndrome presenting with cognitive impairment. *Neurology*.

[39] Meslier N, Lebrun T, Grillier-Lanoir V (1998). A French survey of 3,225 patients treated with CPAP for obstructive sleep apnoea: benefits, tolerance compliance and quality of life. *European Respiratory Journal*.

